# Effects of phenylalanine supplementation on meat fatty acid composition, gut microbiota, and pork volatile organic compound profile in finishing pigs

**DOI:** 10.1186/s40104-026-01496-7

**Published:** 2026-09-12

**Authors:** Mingyang Tan, Zhenglei Shen, Enfa Yan, Boyang Wan, Linjuan He, Qiuyu Zeng, Jingdong Yin

**Affiliations:** 1 State Key Laboratory of Animal Nutrition and Feeding, College of Animal Science and Technology, China Agricultural University, Beijing, 100193 China; 2 Molecular Design Breeding Frontier Science Center of the Ministry of Education (MOE), Beijing, 100193 China

**Keywords:** Gut microbiota, Meat quality, Phenylalanine, Pigs, Volatile organic compounds

## Abstract

**Background:**

Pork quality is a crucial factor influencing consumer purchasing decisions. Previous research has indicated that dietary amino acids such as lysine, arginine, and tryptophan can significantly affect meat quality in finishing pigs. However, the role of phenylalanine, an essential amino acid, in influencing meat quality has not been fully clarified.

**Results:**

In this study, we investigated the effects of increasing dietary phenylalanine/lysine (P/L) ratios on meat quality using castrated finishing barrows. The results showed that dietary P/L ratios of 0.75, 0.90 and 1.05 did not affect carcass traits compared with the control ratio of 0.60 (*P* > 0.05). Pigs fed a diet with a P/L ratio of 0.75 exhibited a significant increase in meat a^*^ value at 45 min postmortem (*P* = 0.01), along with alterations in fatty acid profiles and gut microbiota compositions. PICRUSt2 analysis revealed that the fatty acid metabolic pathway was significantly enriched in predicted functional potential of the gut microbiota under the 0.75 P/L treatment, and *Coprococcus*, *Lachnospiraceae_NK4B4_group*, and *Dialister* were significantly correlated with muscle fatty acid profiles (*P* < 0.05). Furthermore, the PLSR analysis showed that muscle C8:0, C10:0, and C24:1 were negatively correlated with pork volatile organic compounds (VOCs).

**Conclusions:**

This study demonstrated that dietary P/L ratios affected gut microbiota composition in castrated finishing barrows, as well as meat color, fatty acid composition, and free amino acid profiles of pork. Moreover, fatty acids (specifically C8:0, C10:0, and C24:1) correlated with gut microbiota and were associated with differences in the VOC profile between the 0.75 and 0.60 P/L ratio groups.

**Supplementary Information:**

The online version contains supplementary material available at https://doi.org/10.1186/s40104-026-01496-7.

## Background

Pork, one of the most widely consumed meats worldwide, greatly affects consumer preference and eating experience due to its eating quality, thereby influencing purchase decisions [1]. Core indicators for evaluating pork eating quality typically encompass meat color, tenderness, flavor, and juiciness [2]. Currently, studies concerning pork eating quality mainly focus on meat processing technologies while largely overlooking the intrinsic attributes of pork itself. These attributes are influenced by muscle traits such as muscle fiber types, muscle pH levels at 45 min and 24 h postmortem, water-holding capacity, intramuscular fat (IMF) content, fatty acid composition, as well as by complex interactions among these traits. For instance, the postmortem dynamic changes in muscle pH are significantly correlated with water-holding capacity of pork [3]. Meanwhile, IMF content and fatty acid profile, particularly the proportion of polyunsaturated fatty acids (PUFA), directly impact the formation of flavor compounds in cooked pork [4]. Furthermore, there are substantial variations in pork quality traits, especially in drip loss and IMF content, with variations reaching up to 56.8% and 42.9%, respectively. These variations are influenced by breeds, nutritional strategies, management practices, and environmental factors such as seasonal changes in temperature and humidity [5]. Therefore, efficient and consistent production of high-quality pork is a pivotal challenge for ensuring the sustainability of the pork supply chain.

Dietary amino acids significantly influence meat quality in finishing pigs [6]. For example, diets with low standardized ileal digestible (SID) lysine markedly increase IMF content in pork [7, 8]. The optimal dietary valine/isoleucine ratio substantially reduces pork drip loss [9]. In addition, dietary supplementation with arginine and glutamic acid significantly improves pork flavor [10].

There is limited research on the relationship between dietary phenylalanine intake and meat quality in farm animals. Phenylalanine, an aromatic amino acid, has been shown to significantly influence lipid metabolism in the fields of public health and sports medicine. Studies have shown that obese patients have higher plasma levels of phenylalanine and tyrosine [11]. It has been shown that pre-exercise oral phenylalanine administration stimulated fat oxidation by increasing plasma glycerol and glucagon concentrations [12]. In addition, N-lactoyl-phenylalanine, synthesized from lactate and phenylalanine, was shown to function as a novel blood-borne metabolic signaling molecule that aids in reducing body weight [13]. Phenylalanine-derived microbial metabolites, such as 4-hydroxyphenylacetic acid, can inhibit intestinal lipid absorption and metabolism, thereby conferring protection against high-fat diet-induced obesity in mice [14]. Additionally, phenyllactic acid, a phenylalanine metabolite derived from *Lactobacillus*, reduces fat accumulation by activating the intestinal PPAR-γ signaling pathway in mice [15].

Beyond its role in regulating lipid metabolism, dietary phenylalanine supplementation has been shown to reshape the gut microbial structure and alter metabolite profiles in rats [16]. Previous studies have also demonstrated that specific gut bacteria (e.g., *Bacteroides uniformis*, *Lactobacillus reuteri*) directly regulate pork IMF content and systemic fat deposition [4, 17, 18]. Notably, pork IMF content is a key determinant of flavor, juiciness, and tenderness [19]. Moreover, gut microbiota can influence water-holding capacity, shear force, and meat color by modulating muscle oxidative/anti-oxidative balance and postmortem pH dynamics [20, 21].

Considering the close associations between phenylalanine intake, gut microbiota, lipid metabolism, and IMF content, it is plausible that dietary phenylalanine intake may influence meat quality. To date, no studies have examined the effect of dietary phenylalanine supplementation on meat quality traits. Therefore, the present study was designed to investigate the effect of dietary phenylalanine intake on pork quality.

## Materials and methods

### Experimental design

The experiment was performed at the Zhuozhou Animal Testing Base of the Ministry of Agriculture and Rural Affairs Feed Industry Centre (Zhuozhou, Hebei, China). All the procedures performed in this study were approved by the Animal Care and Use Committee of China Agricultural University (ID: AW02015202-1-02). A total of 96 finishing Large White pigs (83.95 ± 0.57 kg) were randomly allocated to four dietary treatments with six replicates per treatment and four pigs (two castrated barrows and two gilts) per replicate. During the 38-day feeding trial, pigs had ad libitum access to feed and water. Experimental diets were formulated to meet or exceed NRC (2012) [22] requirements for 75–135 kg pigs, except that phenylalanine/lysine (P/L) ratios were set at 0.60 (control, NRC recommendation), 0.75, 0.90 and 1.05. Accordingly, pigs weighing 75–100 kg were fed diets with 0.73% SID lysine and containing SID phenylalanine levels of 0.44%, 0.55%, 0.66% and 0.77% for each dietary treatment, and pigs weighing 100–135 kg BW received diets with 0.61% SID lysine and containing SID phenylalanine levels of 0.37%, 0.46%, 0.55% and 0.65% for each dietary treatment. The ingredient composition and nutritional levels of the diets are shown in Table 1.

**Table 1:** The gradient composition and nutrient content of the basal diets for finishing pigs (as fed basis), %

Items	75–100 kg	100–135 kg
Ingredients		
Corn	73.80	79.00
Soybean meal	8.00	4.00
Wheat bran	13.00	12.63
Soybean oil	1.60	1.00
L-Lysine·HCl	0.53	0.47
DL-Methionine	0.04	0.02
L-Threonine	0.19	0.17
L-Tryptophan	0.03	0.03
L-Valine	0.07	0.05
L-Isoleucine	0.07	0.05
L-Phenylalanine	0.00	0.00
L-Alanine	0.34	0.28
Limestone	0.95	0.70
CaHPO_4_	0.55	0.90
NaCl	0.29	0.34
50% Choline chloride	0.03	0.08
Premix^1^	0.50	0.50
Total	100.00	100.00
Analyzed nutrient levels		
Crude protein	12.63	10.94
Lysine	0.75	0.61
Methionine + Cysteine	0.38	0.35
Threonine	0.51	0.45
Isoleucine	0.43	0.35
Leucine	0.99	0.91
Valine	0.55	0.48
Phenylalanine	0.48	0.41
Phenylalanine + Tyrosine	0.80	0.67

^1^The premix provided per kg of diet: Vitamin A, 6,000 IU; Vitamin B₁, 0.96 mg; Vitamin B₆, 2 mg; Vitamin D₃, 2,400 IU; Vitamin E, 20 IU; Vitamin K₃, 2 mg; Vitamin B₂, 4 mg; Vitamin B₁₂, 0.012 mg; Biotin, 0.04 mg; Folic acid, 0.40 mg; Pantothenic acid, 11.1 mg; Niacin, 22 mg; Copper, 120 mg; Iron, 76 mg; Manganese, 12 mg; Zinc, 75 mg; Potassium, 0.24 mg; Selenium, 0.40 mg; Phytase, 100 mg

### Sample collection

At the end of the feeding trial, fecal samples were collected from each pig using sterile tubes, immediately frozen in liquid nitrogen, and stored at −80 °C for subsequent microbial analysis. For microbiota sequencing, one fecal sample from a single castrated barrow was randomly selected from each pen to minimize the potential confounding effect of sex on microbial community composition. Following an overnight fasting, all pigs were transported to the local abattoir. After resting for at least 4 h, pigs were humanely stunned using electricity. After their slaughter weight was recorded, pigs were processed in compliance with standard commercial procedures, including exsanguination, dehairing, and evisceration. Heads, feet, and tails were removed, and the carcasses were split lengthwise [23]. After the weight was recorded, the split carcass was moved to cold storage. The *longissimus thoracis* (LT) muscle was isolated between the 10^th^ and 12^th^ ribs from the left side of each carcass, followed by trimming of adipose and connective tissues. For each treatment, eight castrated barrows (one or two randomly selected from each pen) were used for carcass trait measurement, meat quality evaluation, and chemical composition analyses of free amino acids, fatty acids, and volatile organic compounds (VOCs), ensuring sex consistency across these measurements.

### Carcass and meat quality evaluation

The subsequent evaluation of carcass traits and meat quality was performed according to the methods described previously [5]. The dressing percentage was determined by dividing the hot carcass weight by the slaughter weight. Backfat thickness was measured on the left carcass at the thickest part of the shoulder, 6^th^–7^th^ rib, 10^th^ rib, last rib and lumbar-sacral junction, and the average backfat thickness was calculated as the mean of measurements taken at the shoulder, last rib and lumbar-sacral sites. The loin eye area was determined by length × width × 0.7, while the lean yield index was calculated based on the formula provided by NRC [24]: 50.767 + 0.035 × hot carcass weight (lb) − 8.979 × last-rib backfat (in).

At 45 min and 24 h postmortem, meat color was visually scored according to the National Pork Producer Council (NPPC) standards after a 30-min blooming period. pH values were measured at the same time points using a portable pH meter (Testo 205, Testo AG, Schwarzwald, Germany) in a 4 °C chilled room, and each sample was measured three times. Instrumental color analysis was performed with a Konica Minolta CR-410 colorimeter (Osaka, Japan). The device, equipped with an 8-mm aperture and standardized xenon illumination, was configured with a D65 light source and a 2º observer to determine lightness (L^*^), redness (a^*^) and yellowness (b^*^) values according to the CIE Lab system. Drip loss was measured by suspending *LT* muscle samples at 4 °C for 24 h and calculated by the equation: drip loss (%) = [(initial weight − final weight)/initial weight] × 100. For cooking loss measurement, pre-weighed pork samples were initially immersed in the 75 °C water until the internal temperature of 70 °C was attained. Following equilibration to ambient temperature, samples were reweighed, and cooking loss was quantified as the percentage reduction in mass relative to the initial weight.

To measure the shear force, cylindrical samples with a diameter of 1 cm were taken from cooked pork along the fiber direction. Measurements were performed using a digital tenderness meter (C-LM3B, Tenovo, Harbin, China) by cutting the samples perpendicular to the muscle fiber axis at a speed of 300 mm/min [25]. A total of 10 technical replicates were conducted for each sample and averaged.

Approximately 10 g of LT muscle was sliced and freeze-dried to constant weight in a vacuum freeze-dryer (Freezone 4.5™, Labconco Corp., Kansas City, MO, USA). The sample was weighed before and after freeze-drying to determine the moisture content, which was calculated as the percentage of moisture loss relative to the initial weight. For IMF content, freeze-dried muscle was ground into powder, and IMF was extracted with petroleum ether using a Soxhlet extraction apparatus (Budwi Extraction System B-11, Lausanne, Switzerland). The IMF content was expressed as the percentage of extracted fat relative to the fresh meat weight [26].

### Fatty acid composition

The content of fatty acids was determined according to Xu et al. [9]. Specifically, approximately 150 mg of freeze-dried muscle was weighed into a 10 mL dry glass tube containing 4 mL anhydrous methanol/acetyl chloride (10:1, v/v) mixture, 1 mL n-hexane and 1 mL internal standard (C11:0, 1 mg/mL). The mixture was heated at 80 °C for 3 h in a water bath. After cooling to room temperature, 5 mL of a 70 g/L (7% w/v) potassium carbonate solution was added, followed by centrifugation at 1,000 × *g* for 5 min. The resulting supernatant was passed through a 0.22-µm organic solvent-compatible syringe filter into a GC vial for subsequent chromatographic analysis.

Analysis was performed using an Agilent 6890 gas chromatograph equipped with a flame ionization detector (FID). Separation was achieved on a DB-23 capillary column (60 m × 0.25 mm ×0.25 µm). The injector and detector temperatures were maintained at 260 and 270 °C, respectively. Samples were injected in split mode at a 30:1 ratio, with an injection volume of 1 µL. High-purity helium was used as the carrier gas at a constant flow rate of 2.0 mL/min. The oven temperature was programmed as follows: hold at 140 °C for 5 min, then increase to 240 °C at a rate of 4 °C/min and hold for 15 min.

### Free amino acid analysis

Approximately 300 mg of freeze-dried LT meat was weighed into a 15-mL centrifuge tube. After adding 5 mL methanol/water (8:2, v/v) extraction solvent and 8 µL internal standard (D-Phe, 2.5 mmol/L), the mixture was vortex-mixed and ultrasonicated at ambient temperature for 5 min, followed by a 1-min incubation. This extraction cycle was repeated six times. Samples were then incubated on ice for 2 h and centrifuged at 9,000 × *g* for 10 min at 4 °C to collect the supernatant. The residual pellet was re-extracted with 4 mL methanol/water (8:2, v/v) using identical procedures. The combined supernatants were filtered through 0.1 µm membranes, and 500 µL filtrate was vacuum-dried at 45 °C for 3.5 h. The residue was reconstituted in 100 µL of borate buffer and a 10-µL aliquot was derivatized with AccQ-Tag reagent (Waters, Milford, MA, USA) at 55 °C for 10 min, followed by analysis using a UPLC (ACQUITY UPLC I-Class, Waters, Milford, MA, USA).

### Microbial DNA extraction and 16S rRNA sequencing

Fecal microbial genomic DNA was extracted using the FastPure Stool DNA Isolation Kit (Majorbio, Shanghai, China). DNA purity and concentration were assessed with NanoDrop2000 (Thermo Scientific, Waltham, MA, USA), and the integrity was verified by 1% agarose gel electrophoresis. Amplification of the V3–V4 hypervariable regions employed barcoded primers (forward: 5′-ACTCCTACGGGGAGGCAGCAG-3′, reverse: 5′-GGACTACHVGGTWTCTAAT-3′) under standard PCR conditions. The PCR products were purified with a PCR clean-up kit (Yuhua Biotech, Shanghai, China) and quantified using Qubit 4.0 (Thermo Fisher Scientific, Waltham, MA, USA). Purified amplicons were subjected to paired-end sequencing on Illumina Nextseq2000 (Illumina, San Diego, CA, USA) according to the procedures of Majorbio Bio-Pharm Technology Co., Ltd. (Shanghai, China).

All raw sequences underwent quality control using fastp (version 0.19.6) with Q20 filtering and adapter trimming. Overlapping reads were merged using FLASH (version 1.2.11). Processed sequences were clustered into OTUs at 97% similarity via UPARSE (version 7.1) with dereplication and chimera filtering. Taxonomic annotation was performed using RDP Classifier (version 2.11) against the SILVA 16S rRNA database (version 138) at a 70% confidence threshold. Community composition was analyzed from phylum to genus levels. Functional potential of the microbiota was predicted using PICRUSt2 (version 2.2.0) followed by KEGG pathway enrichment analysis.

### Determination of volatile organic compounds

The content of VOCs was measured as described previously [27]. Fresh meat was ground using a grinder, and 3 g of the homogenate was weighed into a 20-mL headspace vial, followed by the addition of 10 µL of an internal standard solution (10 µg/mL 2-methyl-3-heptanone). The vial was immediately sealed with a silicone septum and crimp cap, and then incubated at 80 °C for 30 min prior to analysis.

Solid-phase microextraction (SPME) was conducted using an automated sampler (Gerstel, Münster, Nordrhein-Westfalen, Germany). After initial incubation, the headspace vials were re-equilibrated at 55 °C for 20 min, followed by volatile extraction for 40 min at 55 °C using an SPME fiber (50/30 µm, DVB/CAR/PDMS, Supelco, Inc., Bellefonte, PA, USA) under continuous agitation. After extraction, the fiber was desorbed at 250 °C for 3 min, with inter-injection fiber conditioning at 270 °C for 10 min.

Volatile compound analysis was performed on a Q-Exactive™ Orbitrap mass spectrometer (Thermo Fisher Scientific, Bremen, Freie Hansestadt Bremen, Germany) coupled to a Trace 1310 GC equipped with an autosampler. Separation was performed using a VF-WAX ms column (60 m × 0.25 mm i.d. × 0.25 µm, Agilent, Santa Clara, CA, USA) with a constant helium flow rate of 1.0 mL/min (99.9999% purity). The temperature program was initiated at 40 °C for 2 min, then ramped at 4 °C/min to 230 °C with a final hold time of 5 min. Mass spectrometry operated in full-scan mode (30–400 *m/z*) at a resolution of 60,000 using electron ionization at 70 eV. The ion source and transfer line temperatures were maintained at 280 and 250 °C, respectively.

GC–MS data acquisition and processing were performed using Xcalibur 4.1 and TraceFinder 4.0 software (Thermo Fisher Scientific, Bremen, Freie Hansestadt Bremen, Germany). VOCs were identified by comparing their mass spectra with those in the in-house reference library and the NIST17 database (version 2.3), along with their linear retention indices. Linear retention indices were calculated using a homologous series of n-alkanes (C_7_–C_40_) under identical analytical conditions and were compared with the literature values to confirm the identified compounds. Aroma compounds in pork were semi-quantified with 2-methyl-3-heptanone as an internal standard.

### Statistical analysis

Carcass traits, meat quality parameters, fatty acids, free amino acids, and VOCs were analyzed using a nested linear mixed model (LMM). In the model, dietary P/L ratio was included as a fixed effect, and pen nested within treatment was included as a random effect. Individual pigs were considered as sampling units, with residual errors treated as random errors. Model parameters were estimated using the restricted maximum likelihood (REML) method. The model for the data was the following equation:

$${Y}_{ijk}=\mu +{T}_{i}+{P}_{j(i)}+{e}_{ijk}$$

where *Y*_*ijk*_ is the observed value of the *k*^th^ pig in the *j*^th^ pen of the *i*^th^ treatment; *μ* is the overall mean; *T*_*i*_ is the fixed effect of the *i*^th^ treatment; *P*_*j(i)*_ is the random effect of the *j*^th^ pen nested within the *i*^th^ treatment; *e*_*ijk*_ is the random residual error at the individual level.

The overall fixed effect of treatment was assessed using the F-test with Type III sums of squares. When the overall effect was significant (*P* < 0.05), pairwise comparisons among treatment means were further performed using the Sidak correction for multiple comparisons to control the family-wise error rate (FWER). In addition, orthogonal polynomial contrasts were applied based on the four concentration gradients to test linear and quadratic effects of dietary P/L ratio on carcass traits, meat quality, fatty acids, and free amino acids. All data are presented as least squares means ± standard error of the mean (LSM ± SEM). All statistical analyses were performed using SPSS 23.0. Statistical significance was set at *P* < 0.05. The quantitative calculation formula of VOCs is as follows:

$$Ca=\frac{Sa\times Cis\times Vis\times 1,000}{Sis\times m}$$

*Ca*: Concentration of VOCs in pork (µg/kg);

*Sa*: Peak area of the target VOCs;

*Sis*: Peak area of 2-methyl-3-heptanone;

*Cis*: Concentration of 2-methyl-3-heptanone (10 µg/mL);

*Vis*: Volume of 2-methyl-3-heptanone added (10 µL);

*m*: Sample mass (g);

1,000: Conversion factor from µg/g to µg/kg.

16S rRNA sequencing data were analyzed on the Majorbio Cloud Platform (https://cloud.majorbio.com). Alpha diversity indices (Chao1, Shannon, Ace, Simpson) were calculated using Mothur v1.30.1. Microbial community similarity was assessed via Principal Coordinate Analysis (PCoA) based on Bray–Curtis dissimilarity, using the vegan v2.5-3 package. PERMANOVA was used to quantify treatment-related variation and its statistical significance. LEfSe analysis identified significantly abundant bacterial taxa (from phylum to genus level) among groups (LDA score > 2) and the resulting *P* values were adjusted for multiple testing using the Benjamini–Hochberg procedure (adjusted *P* < 0.05).

Principal Component Analysis (PCA) and Orthogonal Partial Least Squares Discriminant Analysis (OPLS-DA) of pork VOCs were performed using the MetaboAnalyst 6.0 online platform (https://www.metaboanalyst.ca/). Pearson correlation analysis was conducted in R (version 4.4.3), and the resulting *P*-values were adjusted for multiple testing using the Benjamini–Hochberg procedure. Bar plots were generated using GraphPad Prism 8.0 (GraphPad Software, La Jolla, CA, USA). Partial Least Squares Regression (PLSR) analysis was performed with XLSTAT (Addinsoft Inc., New York, NY, USA). Mantel tests were executed in R (version 4.4.3) using the vegan and linkET packages, with dplyr for data preprocessing and ggplot2 for visualization.

## Results

### Meat quality

Dietary P/L ratios did not significantly affect carcass traits in finishing pigs (Table 2). However, increasing dietary P/L ratios quadratically improved pork pH_45min_ and a^*^_45min_ (*P* = 0.01), while linearly reducing 24 h meat color scores (*P* = 0.03). Notably, pigs fed a diet with a 0.75 P/L ratio had the highest pH_45min_, and the greatest a^*^_45min_ values among all pigs (Table 3).

**Table 2:** Effects of dietary P/L ratio on carcass traits of castrated finishing barrows (*n* = 8)

Items	Dietary P/L ratio				SEM	*P* value		
	**0.60**	**0.75**	**0.90**	**1.05**		**Anova**	**Linear**	**Quadratic**
Live body weight, kg	132.92	133.26	132.86	133.05	2.51	1.00	0.86	0.99
Carcass weight, kg	102.71	101.43	99.94	101.56	1.83	0.76	0.55	0.43
Carcass length, cm	84.89	82.69	86.95	83.55	1.85	0.41	0.86	0.65
Dressing percentage, %	77.49	76.34	75.28	76.24	0.84	0.34	0.15	0.16
Back fat depth, mm								
Shoulder fat thickness	42.92	42.03	36.00	39.37	1.99	0.09	0.07	0.29
Last rib fat thickness	29.87	26.04	26.60	26.97	1.64	0.37	0.28	0.21
Lumbosacral fat thickness	15.82	15.93	13.17	14.94	0.95	0.18	0.19	0.40
6^th^ to 7^th^ rib fat thickness	28.06	25.45	25.79	27.19	1.97	0.77	0.78	0.34
10^th^ rib fat thickness	23.73	23.69	19.93	21.88	1.77	0.39	0.25	0.58
Average back-fat depth^a^	30.20	27.85	25.30	27.10	1.35	0.10	0.06	0.14
Loin eye area, cm^2^	36.50	33.92	37.23	36.53	1.24	0.27	0.54	0.46
Fat-free lean index	48.67	49.23	48.33	49.10	0.67	0.77	0.90	0.88
Fat-free lean percentage, %	46.25	45.84	47.34	46.83	0.81	0.59	0.38	0.95

^a^Average back-fat depth = (Shoulder fat thickness + Last rib fat thickness + Lumbosacral fat thickness)/3

**Table 3:** Effects of dietary P/L ratio on meat quality of castrated finishing barrows (*n* = 8)

Items	Dietary P/L ratio				SEM	*P* value		
	**0.60**	**0.75**	**0.90**	**1.05**		**Anova**	**Linear**	**Quadratic**
pH_45min_	5.81^b^	6.09^a^	5.86^ab^	5.79^b^	0.05	< 0.01	0.24	0.01
pH_24h_	5.44	5.49	5.39	5.43	0.03	0.05	0.24	0.94
Flesh color score_45min_	3.06^ab^	3.56^a^	2.88^ab^	2.69^b^	0.20	0.03	0.06	0.10
Flesh color score_24h_	2.63^ab^	3.07^a^	2.25^b^	2.38^b^	0.16	< 0.01	0.03	0.31
L^*^_45min_	47.36	47.69	46.20	48.12	1.04	0.60	0.87	0.45
a^*^_45min_	15.32^b^	16.24^a^	15.55^ab^	15.50^ab^	0.17	0.02	0.86	0.01
b^*^_45min_	2.96	2.96	3.13	3.31	0.22	0.65	0.22	0.74
L^*^_24h_	53.44	52.77	52.27	52.54	0.82	0.77	0.39	0.57
a^*^_24h_	16.67	16.51	16.08	16.03	0.44	0.66	0.24	0.77
b^*^_24h_	8.11	7.99	7.43	7.44	0.39	0.48	0.10	0.74
Drip loss, %	2.60	2.48	1.16	2.95	0.55	0.13	0.91	0.09
Cooking loss, %	27.56	27.56	28.48	26.98	0.81	0.62	0.82	0.36
Shear force, N	87.30	92.48	90.22	89.74	3.44	0.77	0.54	0.39
Marbling score	2.10	2.20	2.06	2.18	0.13	0.84	0.90	0.82
Moisture, %	73.82	74.03	73.27	73.21	0.42	0.44	0.18	0.75
Intramuscular fat, %	2.97	3.38	3.12	3.41	0.40	0.84	0.51	0.88

^a,^^b^Within a row, means without a common superscript differ significantly (*P* < 0.05)

### Free amino acid content in pork

Free amino acids were classified into umami, sweet, bitter, and other groups as described previously [23]. As shown in Table 4, as the P/L ratio increased, Ser, Gly, Thr, Ala, Asp, His, Met, Val, Ile, Phe, Pro, Cys, and Tyr exhibited significant linear increases (*P* < 0.05), whereas Lys showed a linear decrease (*P* = 0.03). In addition, several amino acids, including Ser, Arg, Gly, Thr, His, Val, and Tyr, exhibited significant quadratic responses (*P* < 0.05). Compared to the control (0.60 ratio), the 1.05 ratio led to a significant increase in most sweet (Ser, Gly, Thr), umami (Asp), and bitter (His, Met, Val, Phe) amino acids, as well as Pro and Tyr (*P* < 0.05).

**Table 4:** Effects of dietary P/L ratio on free amino acid profile in castrated finishing barrows (*n* = 8), nmol/g

Items	Dietary P/L ratio				SEM	*P* value		
	**0.60**	**0.75**	**0.90**	**1.05**		**Anova**	**Linear**	**Quadratic**
Sweet								
Serine (Ser)	134.53^b^	155.58^b^	139.22^b^	250.272^a^	17.96	< 0.01	0.01	0.02
Arginine (Arg)	67.17	65.99	52.95	79.80	6.48	0.08	0.34	0.04
Glycine (Gly)	408.07^b^	418.46^b^	439.35^b^	760.96^a^	59.06	< 0.01	0.01	0.02
Threonine (Thr)	129.20^b^	148.13^b^	139.18^b^	248.10^a^	8.05	< 0.01	0.01	0.01
Alanine (Ala)	2,761.93^ab^	2,416.28^b^	3,016.58^a^	3,021.72^a^	106.52	< 0.01	0.01	0.11
Umami								
Aspartic acid (Asp)	28.05^b^	34.30^b^	38.34^b^	59.39^a^	4.69	< 0.01	0.01	0.07
Glutamic acid (Glu)	472.74	576.71	539.20	472.27	66.76	0.62	0.86	0.15
Bitter								
Histidine (His)	201.70^a^	203.29^a^	237.75^b^	295.13^c^	4.68	< 0.01	0.01	0.01
Methionine (Met)	393.54^b^	541.20^ab^	425.54^ab^	652.24^a^	54.93	0.02	0.03	0.43
Valine (Val)	126.74^b^	121.13^b^	112.71^b^	223.71^a^	6.93	< 0.01	0.01	0.01
Isoleucine (Ile)	243.17^ab^	229.65^b^	247.43^ab^	276.48^a^	11.29	0.05	0.03	0.07
Leucine (Leu)	540.95	513.89	535.03	590.97	23.54	0.16	0.13	0.12
Phenylalanine (Phe)	1,841.86^b^	1,931.59^b^	2,002.68^ab^	2,407.40^a^	112.19	< 0.01	0.01	0.17
Others								
Proline (Pro)	234.74^b^	301.32^bc^	351.37^ac^	416.50^a^	27.04	< 0.01	0.01	0.85
Cysteine (Cys)	44.73	66.18	87.87	117.70	19.41	0.10	0.01	0.84
Lysine (Lys)	911.36	896.98	756.82	699.64	81.06	0.20	0.03	0.78
Tyrosine (Tyr)	152.04^b^	156.63^b^	158.90^b^	227.33^a^	6.41	< 0.01	0.01	0.01
Tryptophan (Trp)	143.28	150.84	148.02	164.93	6.39	0.13	0.07	0.50

^a,^^b^Within a row, means without a common superscript differ significantly (*P* < 0.05)

### Fatty acid composition in pork

As shown in Table 5, significant linear responses to increasing P/L ratio were observed for C15:0 (*P* < 0.01), C16:1 (*P* < 0.01), C17:0 (*P* < 0.01), and C20:0 (*P* = 0.02). Notably, several fatty acids, including C8:0, C10:0, C12:0, C14:1, C18:1n9, C18:2n6, C20:1, C21:0, C20:4n6, C22:6n3, C23:0, C24:1, PUFA, and n-6 PUFA, along with the n-6/n-3 ratio, exhibited significant quadratic responses (*P* < 0.05). Furthermore, increasing the dietary P/L ratio from 0.60 to 0.75 significantly elevated the proportions of C8:0, C10:0, C22:6n3, and C24:1 in pork (*P* < 0.05).

**Table 5:** Effects of dietary P/L ratio on fatty acid profiles in castrated finishing barrows (*n* = 8), % of total fatty acids

Items	Dietary P/L ratio				SEM	*P* value		
	**0.60**	**0.75**	**0.90**	**1.05**		**Anova**	**Linear**	**Quadratic**
C8:0	0.023^b^	0.038^a^	0.029^ab^	0.029^ab^	0.003	< 0.01	0.40	0.01
C10:0	0.138^b^	0.171^a^	0.148^ab^	0.144^ab^	0.007	0.02	0.86	0.02
C12:0	0.091	0.099	0.096	0.090	0.003	0.19	0.69	0.03
C14:0	1.358	1.284	1.325	1.254	0.040	0.31	0.11	0.81
C14:1	0.025^ab^	0.029^a^	0.029^a^	0.023^b^	0.001	< 0.01	0.27	< 0.01
C15:0	0.035^b^	0.044^ab^	0.040^ab^	0.048^a^	0.003	0.02	< 0.01	0.78
C16:0	25.669	25.397	25.777	25.447	0.310	0.80	0.81	0.81
C16:1	3.663^a^	3.524^ab^	3.672^a^	3.107^b^	0.109	< 0.01	< 0.01	0.06
C17:0	0.187^b^	0.229^ab^	0.219^ab^	0.272^a^	0.019	0.04	< 0.01	0.67
C18:0	12.855	13.654	13.372	13.691	0.302	0.22	0.11	0.38
C18:1n9c	44.551	42.581	42.661	43.565	0.719	0.21	0.39	0.04
C18:2n6c	6.721	8.301	8.105	7.313	0.523	0.14	0.50	0.03
C18:3n3	0.224^ab^	0.210^b^	0.253^a^	0.220^ab^	0.010	0.03	0.52	0.36
C20:0	0.234^ab^	0.241^a^	0.211^b^	0.225^ab^	0.006	< 0.01	0.02	0.65
C20:1	0.781	0.706	0.722	0.812	0.029	0.05	0.41	< 0.01
C21:0	0.334	0.438	0.379	0.350	0.028	0.06	0.95	0.02
C20:3n6	0.230	0.303	0.270	0.238	0.028	0.28	0.94	0.08
C20:4n6	1.232	1.861	1.550	1.419	0.180	0.11	0.76	0.04
C20:3n3	0.059	0.044	0.058	0.069	0.009	0.27	0.25	0.15
C20:5n3	0.045	0.062	0.055	0.052	0.006	0.29	0.60	0.12
C22:0	0.057	0.078	0.070	0.073	0.007	0.18	0.20	0.15
C22:1n9	0.241	0.298	0.281	0.328	0.030	0.27	0.06	0.86
C22:6n3	0.041^b^	0.070^a^	0.056^ab^	0.054^ab^	0.006	0.02	0.42	0.02
C23:0	0.047	0.080	0.063	0.053	0.009	0.09	0.98	0.03
C24:0	0.063	0.080	0.066	0.072	0.008	0.51	0.71	0.47
C24:1	0.053^b^	0.086^a^	0.069^ab^	0.066^ab^	0.007	0.04	0.49	0.02
SFA	41.100	41.832	41.814	41.805	0.545	0.73	0.41	0.35
MUFA	49.305	47.198	47.429	47.882	0.715	0.19	0.27	0.06
PUFA	8.549	10.851	10.346	9.365	0.719	0.13	0.55	0.03
n-3PUFA	0.366	0.387	0.421	0.395	0.019	0.26	0.19	0.26
n-6PUFA	8.183	10.465	9.925	8.970	0.703	0.13	0.57	0.03
n-6/n-3	22.278^b^	26.419^a^	22.941^ab^	22.744^ab^	0.962	0.03	0.83	0.01
PUFA/SFA	0.209	0.262	0.248	0.226	0.019	0.24	0.68	0.05

^a,^^b^Within a row, means without a common superscript differ significantly (*P* < 0.05)

### Microbial community profiles

**Fig. 1:**
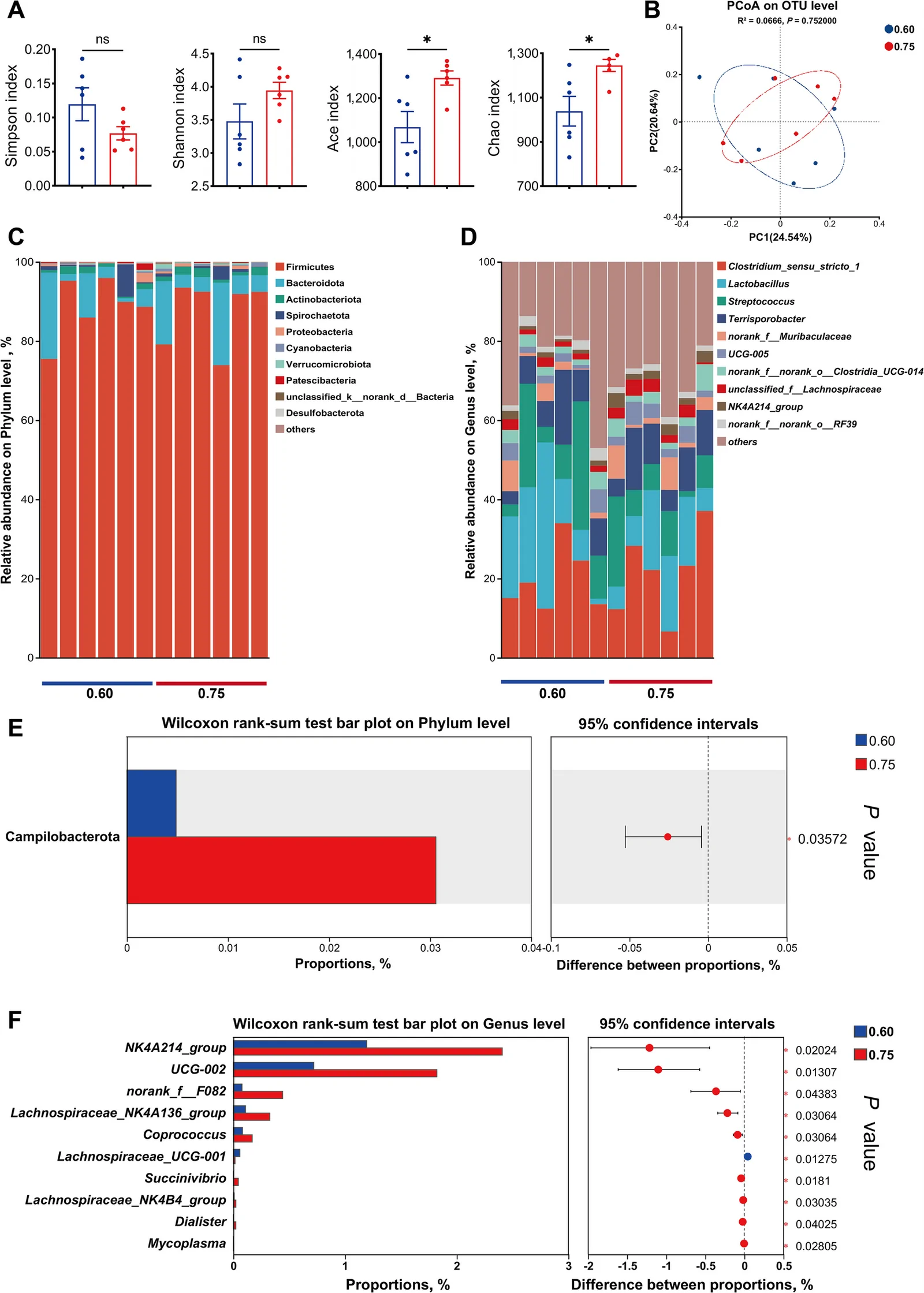
Effects of dietary P/L ratio on fecal microbiota in castrated finishing barrows. **A** Alpha diversity indices. **B** Beta diversity analysis. Microbial composition at phylum (**C**) and genus (**D**) levels, and differentially abundant phyla (**E**) and genera (**F**) of fecal microbiota between the 0.60 and 0.75 groups. 0.60, pigs fed diet with P/L ratio = 0.60; 0.75, pigs fed diet with P/L ratio = 0.75. ^*^P < 0.05

As shown in Table S1, increasing dietary ratio of P/L quadratically increased Ace index (*P* = 0.02), Chao index (*P* = 0.01), and Simpson index (*P* = 0.04) of the gut microbiota in finishing pigs. Particularly, pigs fed a diet with a 0.75 P/L ratio had the highest Ace index and Chao index compared to those receiving the other three dietary treatments (Table S1, Fig. 1A). However, the dietary ratio of P/L did not alter β-diversity of gut microbiota (Fig. 1B, Fig. S1). Firmicutes, Bacteroidota, and Actinobacteriota were the dominant phyla in pigs receiving diets of both P/L ratios of 0.60 and 0.75 (Fig. 1C), and top 10 genera were listed in Fig. 1D. The abundance of Campylobacterota was significantly enhanced in pigs fed a diet with 0.75 P/L ratio compared to control pigs fed a 0.60 P/L ratio diet (Fig. 1E). Furthermore, the abundance of *NK4A214_group*, *UCG-002*, *norank_f__F082*, *Lachnospiraceae_NK4A136_group*, *Coprococcus*, *Succinivibrio*, *Lachnospiraceae_NK4B4_group*, *Dialister*, and *Mycoplasma* were significantly higher in pigs fed the 0.75 P/L ratio diet compared to those on the 0.60 P/L ratio diet. Conversely, the abundance of *Lachnospiraceae_UCG-001* was significantly lower (Fig. 1F).

### Correlation analysis between gut microbiota and muscle fatty acids

**Fig. 2:**
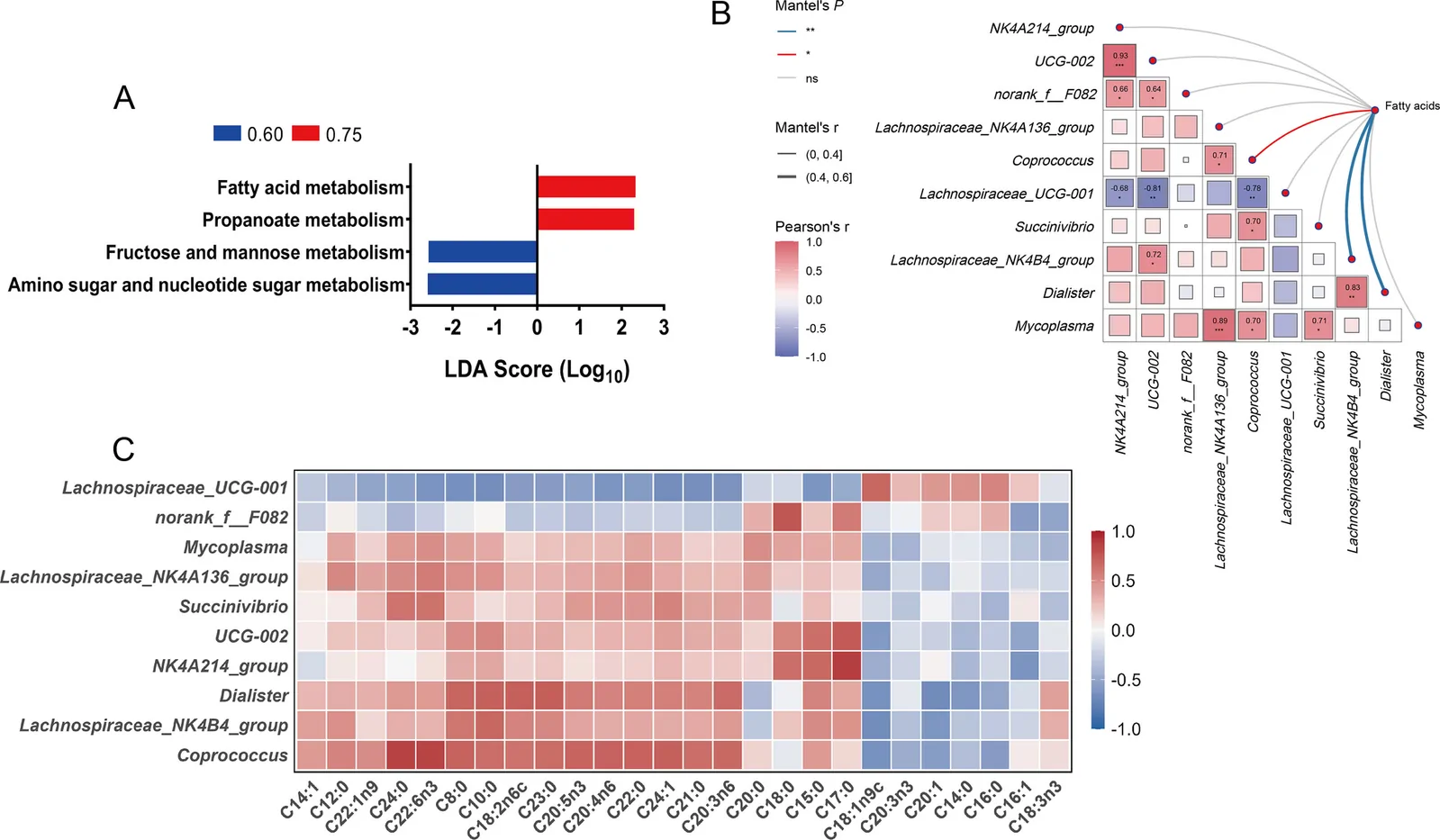
Correlation between gut microbiota and fatty acid profiles in castrated finishing barrows. **A** PICRUSt2 function prediction of gut microbiota. Mantel analysis (**B**) and Pearson correlation analysis (**C**) between muscle fatty acids and differential genera. ^***^P < 0.001; ^**^P < 0.01; ^*^P < 0.05

PICRUSt2 functional prediction revealed that distinct biological processes were involved in the gut microbiota of pigs fed the diets with P/L ratios of 0.60 and 0.75. In pigs fed a diet with a 0.60 P/L ratio, significantly enriched pathways included Fructose and mannose metabolism, as well as Amino sugar and nucleotide sugar metabolism. In contrast, pigs fed a diet with a 0.75 P/L ratio had significantly enriched pathways that included Fatty acid metabolism and Propanoate metabolism (Fig. 2A). In this case, we further examined relationships between pork fatty acid profile and differentially abundant microbial genera. Mantel test demonstrated significant correlations of *Coprococcus*, *Lachnospiraceae_NK4B4_group*, and *Dialister* with muscle fatty acid profiles (*P* < 0.05, Fig. 2B). Furthermore, Pearson correlation analysis confirmed that the proportions of C8:0, C10:0, C22:6n3, and C24:1 were positively correlated with the abundances of *Coprococcus*, *Lachnospiraceae_NK4A136_group*, and *Dialister* (Fig. 2C).

### Volatile organic compounds in pork

**Fig. 3:**
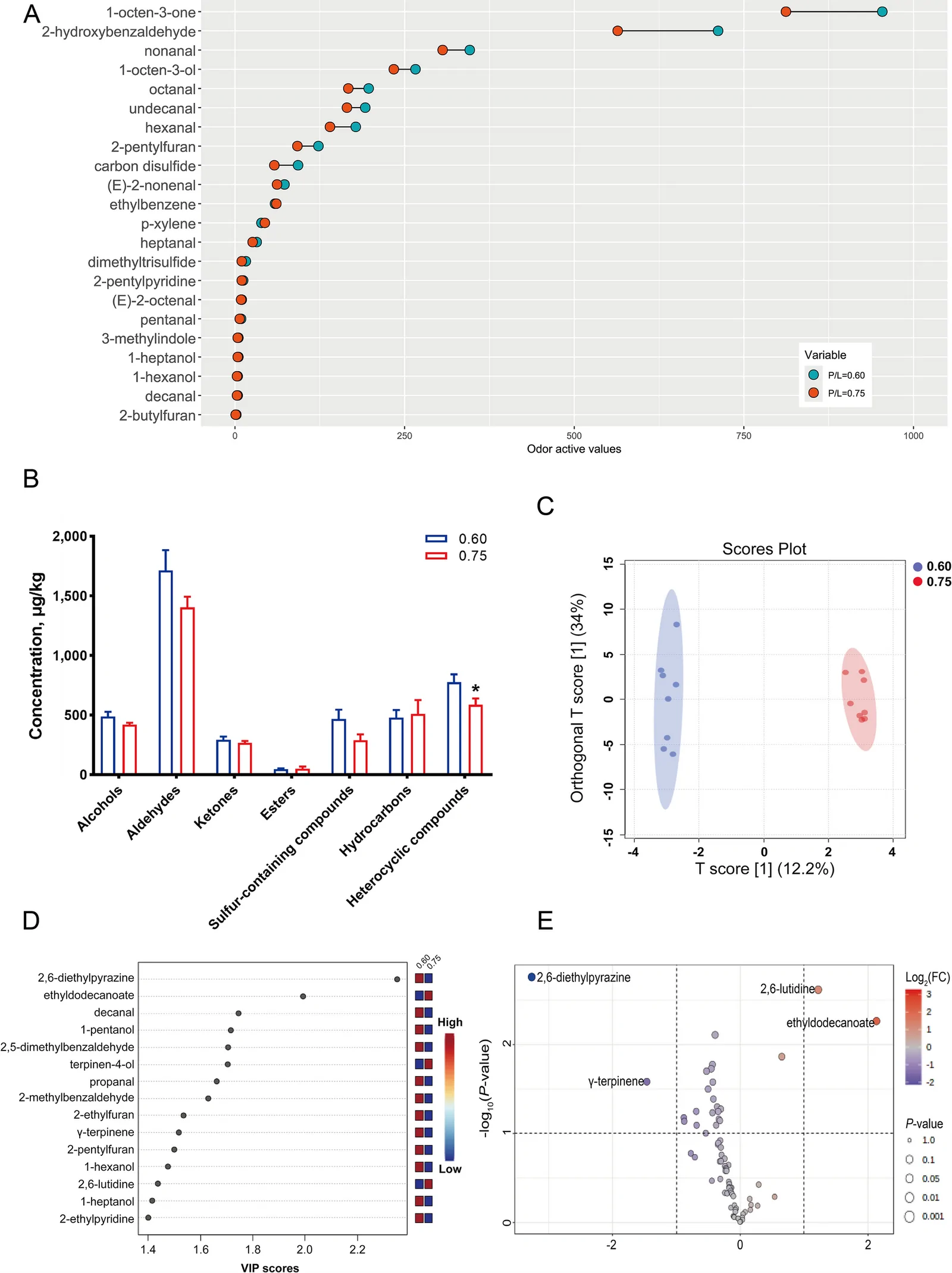
Effects of dietary P/L ratio on volatile organic compounds in castrated finishing barrows. **A** The volatile organic compounds with odor activity value (OAV) > 1. **B** The effect on volatile organic compounds classes. **C** Orthogonal Projections to Latent Structures Discriminant Analysis (OPLS-DA) score plot. **D **and **E** Key volatile compounds screening: (**D**) Variable Importance in Projection (VIP) > 1 (based on OPLS-DA), (**E**) |Log_2_(FC)|> 1. FC, fold change. ^*^P < 0.05

Fatty acids serve as key precursors of VOCs in pork. Among all treatment groups, pigs fed diets with a P/L ratio of 0.75 represented the most pronounced difference in fatty acid composition relative to the control (dietary P/L = 0.60). Consequently, pork samples from these two groups were selected for comparative VOC profiling. As shown in Table 6, a total of 76 VOCs belonging to 7 chemical families were identified and semi-quantified, including 6 alcohols, 4 sulfur-containing compounds, 21 aldehydes, 7 hydrocarbons, 10 ketones, 19 heterocyclic compounds, and 9 esters. An odor activity value (OAV) analysis identified 22 VOCs with OAV > 1 in both groups (Table 7 and Fig. 3A).

**Table 6:** Effects of dietary P/L ratio on volatile organic compounds in cooked *longissimus thoracis* muscle of castrated finishing barrows (*n* = 8), μg/kg

**Compound**	**Linear retention index**		**Dietary P/L ratio**		**SEM**	***P* value**	**Identification**^3^
	**Calculated**^1^	**Literature**^2^	**0.60**	**0.75**			
Alcohols							
1-Butanol	1,132	1,150	0.49	0.47	0.05	0.72	MS, LRI
1-Pentanol	1,235	1,253	39.98^a^	29.18^b^	2.88	0.02	MS, LRI
1-Hexanol	1,337	1,355	21.55	16.76	1.58	0.05	MS, LRI
1-Octen-3-ol	1,433	1,451	398.65	351.13	26.55	0.23	MS, LRI
1-Heptanol	1,439	1,453	25.31	19.91	1.84	0.06	MS, LRI
Terpinen-4-ol	1,593	1,602	0.31^a^	0.48^b^	0.05	0.05	MS, LRI
Sulfur-containing compounds							
Carbon disulfide	724	730	462.72	288.24	65.54	0.08	MS, LRI
Dimethyldisulfide	1,065	1,071	0.92	0.62	0.22	0.36	MS, LRI
Dimethyltrisulfide	1,376	1,386	1.56	0.97	0.34	0.25	MS, LRI
Methional	1,447	1,458	0.79	0.88	0.10	0.54	MS, LRI
Aldehydes							
Propanal	783	801	1.25^a^	0.87^b^	0.10	0.02	MS, LRI
Butanal	867	883	0.91	0.78	0.08	0.25	MS, LRI
3-Methylbutanal	912	923	0.48	0.39	0.04	0.12	MS, LRI
Pentanal	972	969	109.80	88.96	6.98	0.05	MS, LRI
Hexanal	1,075	1,077	889.14	698.05	84.58	0.13	MS, LRI
Heptanal	1,176	1,182	90.58	73.98	8.20	0.17	MS, LRI
(E)-2-Hexenal	1,213	1,230	1.47	1.32	0.13	0.44	MS, LRI
Octanal	1,283	1,298	116.32	98.57	10.07	0.23	MS, LRI
(E)-2-Heptenal	1,318	1,335	23.34	20.62	1.98	0.35	MS, LRI
3-Methyl-2-butenal	1,196	1,215	0.78	0.63	0.11	0.33	MS, LRI
Nonanal	1,386	1,402	380.08	336.82	36.05	0.41	MS, LRI
(E)-2-Octenal	1,424	1,430	30.61	25.83	2.46	0.19	MS, LRI
Decanal	1,493	1,508	12.08^a^	8.91^b^	0.75	0.01	MS, LRI
3-Methylbenzaldehyde	1,613	1,626	0.21	0.19	0.02	0.41	MS, LRI
2,5-Dimethylbenzaldehyde	1,689	1,683	7.76^a^	5.74^b^	0.53	0.02	MS, LRI
(E)-2-Nonenal	1,531	1,534	13.81	11.69	1.99	0.46	MS, LRI
4-Pentylbenzaldehyde	2,000	2,003	7.01	6.72	0.90	0.82	MS, LRI
Undecanal	1,599	1,616	23.08	19.85	1.91	0.25	MS, LRI
2-Methylbenzaldehyde	1,618	1,632	0.54^a^	0.40^b^	0.04	0.03	MS, LRI
2-Hydroxybenzaldehyde	1,664	1,672	2.22	1.76	0.17	0.07	MS, LRI
3-Thiophenecarboxaldehyde	1,674	1,677	0.75	0.61	0.07	0.14	MS, LRI
Hydrocarbons							
p-Xylene	1,135	1,138	281.97	345.11	77.58	0.58	MS, LRI
dl-Limonene	1,194	1,211	1.05	1.27	0.22	0.49	MS, LRI
γ-Terpinene	1,247	1,244	0.22^a^	0.08^b^	0.04	0.03	MS, LRI
Mesitylene	1,240	1,251	1.18	0.73	0.15	0.06	MS, LRI
Toluene	1,034	1,049	44.18	39.14	4.12	0.40	MS, LRI
Ethylbenzene	1,119	1,129	129.78	139.08	30.35	0.83	MS, LRI
Styrene	1,252	1,270	8.64	8.28	2.36	0.92	MS, LRI
Ketones							
2-Heptanone	1,176	1,190	24.08	23.52	3.16	0.90	MS, LRI
6-Methyl-2-heptanone	1,228	1,245	4.93	4.48	0.43	0.46	MS, LRI
3-Octanone	1,246	1,264	15.06	13.80	1.23	0.48	MS, LRI
2-Octanone	1,275	1,293	6.28	5.83	0.47	0.51	MS, LRI
1-Octen-3-one	1,293	1,310	2.86	2.44	0.26	0.26	MS, LRI
2,3-Octanedione	1,311	1,323	234.12	210.45	19.59	0.41	MS, LRI
3-Octen-2-one	1,400	1,417	3.57	3.35	0.32	0.64	MS, LRI
2-Decanone	1,487	1,500	1.24	1.06	0.11	0.255	MS, LRI
4-Octanone	1,221	1,234	0.26	0.25	0.03	0.76	MS, LRI
2-Pentadecanone	2,011	2,019	0.41	0.37	0.04	0.43	MS, LRI
Heterocyclic compounds							
2,3,5-Trimethylfuran	1,047	1,038	1.92	2.21	0.67	0.77	MS, LRI
2-n-Octylfuran	1,529	1,520	6.76	5.08	0.78	0.16	MS, LRI
2-Ethylfuran	945	960	8.93^a^	6.30^b^	0.78	0.03	MS, LRI
2-Butylfuran	1,123	1,142	7.61	6.76	0.65	0.37	MS, LRI
2-Pentylfuran	1,225	1,241	715.15^a^	532.99^b^	57.55	0.05	MS, LRI
2-Hexylfuran	1,325	1,321	1.59	0.90	0.31	0.15	MS, LRI
2-Heptylfuran	1,427	1,444	1.83	1.21	0.24	0.10	MS, LRI
2,6-Lutidine	1,241	1,254	0.18^a^	0.41^b^	0.05	< 0.01	MS, LRI
2-Ethylpyridine	1,274	1,278	0.62	0.46	0.06	0.06	MS, LRI
3-Ethylpyridine	1,374	1,377	0.16	0.13	0.01	0.08	MS, LRI
2-Pentylpyridine	1,566	1,584	7.42	5.95	0.53	0.07	MS, LRI
2,6-Diethylpyrazine	1,437	1,432	1.46^a^	0.15^b^	0.24	< 0.01	MS, LRI
2-Acetyl-pyrrole	1,953	1,972	1.05	0.55	0.22	0.13	MS, LRI
2-Pentylthiophene	1,454	1,473	17.08	17.31	2.31	0.95	MS, LRI
2-Ethylthiophene	1,167	1,179	0.52	0.52	0.06	0.99	MS, LRI
2-Butylthiophene	1,352	1,352	0.69	0.56	0.07	0.21	MS, LRI
2-Hexylthiophene	1,559	1,564	0.32	0.31	0.04	0.88	MS, LRI
Indole	2,427	2,445	0.75	0.59	0.07	0.14	MS, LRI
3-Methylindole	2,474	2,492	0.61	0.53	0.08	0.47	MS, LRI
Esters							
Methyl nonanoate	1,484	1,491	0.13	0.13	0.01	1.00	MS, LRI
Ethyldodecanoate	1,834	1,848	0.05^a^	0.23^b^	0.05	0.03	MS, LRI
Methyldecanoate	1,587	1,593	0.12	0.12	0.02	0.98	MS, LRI
Methyldodecylate	1,791	1,804	0.14	0.17	0.02	0.38	MS, LRI
Methyl-n-tetradecanoate	1,999	2,005	0.33	0.27	0.06	0.48	MS, LRI
Methylpentadecanoate	2,103	2,108	0.09	0.08	0.02	0.86	MS, LRI
Methylhexadecanoate	2,206	2,222	20.03	29.10	9.57	0.51	MS, LRI
Methylheptadecanoate	2,310	2,309	0.91	0.86	0.25	0.87	MS, LRI
Methyl stearate	2,412	2,418	24.24	17.81	4.60	0.34	MS, LRI

^1^Calculated data based on a homologous series of n-alkanes (C7–C40)

^2^Data obtained from library: IAS Centrelab Flavor Database and Mainlib

^3^MS, identified using MS spectra; LRI, identified using the linear retention index

^a,b^Different letters in the same row represent significant differences (*P* < 0.05)

**Table 7:** Volatile organic compounds in cooked pork with odor activity value (OAV) > 1 (*n* = 8), μg/kg

Compound	Thresholds^a^	Dietary P/L ratio		SEM	*P* value	Odor description
		**0.60**	**0.75**			
Hexanal	5^b^	889.14	698.05	84.58	0.13	Green, fresh grass
2-Pentylfuran	5.8^b^	715.15^e^	532.99^f^	57.55	0.05	Green bean, butter
Carbon disulfide	5^b^	462.72	288.24	65.54	0.08	Sulfury, fruity, burnt
1-Octen-3-ol	1.5^b^	398.65	351.13	26.55	0.23	Mushroom
Nonanal	1.1^b^	380.08	336.82	36.05	0.41	Citrus, green, citronella grass
p-Xylene	7.5^c^	281.97	345.11	77.58	0.58	Phenolic
Ethylbenzene	2.2^b^	129.78	139.08	30.35	0.83	Floral
Octanal	0.59^b^	116.32	98.57	10.07	0.23	Green, citrus, lemon
Pentanal	12^b^	109.80	88.96	6.98	0.05	Green
Heptanal	2.8^b^	90.58	73.98	8.20	0.17	Green, aldehyde
(E)-2-Octenal	3^b^	30.61	25.83	2.46	0.19	Green, floral
1-Heptanol	5.4^b^	25.31	19.91	1.84	0.06	Floral
Undecanal	0.12^b^	23.08	19.85	1.91	0.25	Floral, green, mild
1-Hexanol	5.6^b^	21.55	16.76	1.58	0.05	Green
(E)-2-Nonenal	0.19^b^	13.81	11.69	1.99	0.46	Fatty, paper
Decanal	3.0^b^	12.08^e^	8.91^f^	0.75	0.01	Fatty, rancid, meaty, burnt
2-Butylfuran	5^b^	7.61	6.76	0.65	0.37	Green, sweet
2-Pentylpyridine	0.6^b^	7.42	5.95	0.53	0.07	Burnt, fatty
1-Octen-3-one	0.003^b^	2.86	2.44	0.26	0.26	Mushroom
2-Hydroxybenzaldehyde	0.00312^d^	2.22	1.76	0.17	0.07	/
Dimethyltrisulfide	0.1^b^	1.56	0.97	0.34	0.25	Sulfury, meaty
3-Methylindole	0.13^b^	0.61	0.53	0.08	0.47	Mothball-like

^a^Odor thresholds in water from the literature

^b^Sohail et al. [28]

^c^Liu et al. [29]

^d^van Gemert [30]

^e,f^Different letters in the same row represent significant differences (*P* < 0.05)

A P/L ratio of 0.75 in the diet resulted in significantly lower levels of heterocyclic compounds in pork compared to a ratio of 0.60 (Fig. 3B). The OPLS-DA revealed distinct differences in the VOC profiles of pork from pigs fed diets with P/L ratios of 0.60 and 0.75 (Fig. 3C), and Permutation testing (1,000 permutations) validated the OPLS-DA model (R^2^Y = 0.996, Q^2^ = 0.654; both *P* < 0.05 compared with permuted data), confirming that the model was robust and not overfitted (Fig. S2A). The top 10 VOCs ranked by variable importance in projection (VIP) values were shown in Fig. 3D. Based on the Student's two-tailed *t*-test and OPLS-DA, 12 VOCs were identified as being significantly affected by the dietary P/L ratio (*P* < 0.05 and VIP > 1; Table 8). Among them, 2,6-diethylpyrazine and γ-terpinene concentrations decreased the most in pork from pigs fed a diet with a P/L ratio of 0.75 compared to those on a 0.60 P/L ratio diet (Log_2_(FC) < −1), while 2,6-lutidine and ethyldodecanoate increased the most (Log_2_(FC) > 1; Fig. 3E). Notably, among these 22 key VOCs, concentrations of 2-pentylfuran (*P* = 0.041, VIP = 1.50) and decanal (*P* = 0.008, VIP = 1.75) in pork were lower in pigs fed a diet with a 0.75 P/L ratio than in those on the 0.60 P/L ratio diet (Table 8).

**Table 8:** Volatile organic compounds in cooked *longissimus thoracis* muscle of castrated finishing barrows were significantly affected by dietary P/L ratio (*n* = 8), μg/kg

Compound	Dietary P/L ratio		SEM	*P* value^a^	VIP^b^	Odors description^c^
	**0.60**	**0.75**				
Propanal	1.25	0.87	0.10	0.02	1.66	Caramel, sweet^d^
2,6-Lutidine	0.18	0.41	0.05	< 0.01	1.44	Bitterness^e^
2,6-Diethylpyrazine	1.46	0.15	0.24	< 0.01	2.35	/
1-Pentanol	39.98	29.18	2.88	0.02	1.72	Green, fruity^d^
γ-Terpinene	0.22	0.08	0.04	0.03	1.52	Gasoline, turpentine^d^
2-Ethylfuran	8.93	6.30	0.78	0.03	1.54	Fruity, floral^d^
2-Pentylfuran	715.15	532.99	57.55	0.05	1.50	Green bean, butter^d^
Decanal	12.08	8.91	0.75	0.01	1.75	Fatty, rancid, meaty, burnt^d^
2,5-Dimethylbenzaldehyde	7.76	5.74	0.53	0.02	1.71	Spicy, floral^d^
2-Methylbenzaldehyde	0.54	0.40	0.04	0.03	1.63	Hairdresser’s ^d^
Ethyldodecanoate	0.05	0.23	0.05	0.03	1.99	Wax, fruity, fatty^d^
Terpinen-4-ol	0.31	0.48	0.05	0.05	1.70	Roasted, woody^d^

^a^Changes with *P* < 0.05 were considered significant

^b^VIP, variable importance in the projection

^c^The odor description of the compound from the literature

^d^Sohail et al. [28]

^e^van Gemert [30]

### Correlation analysis between fatty acids and VOCs in pork

**Fig. 4:**
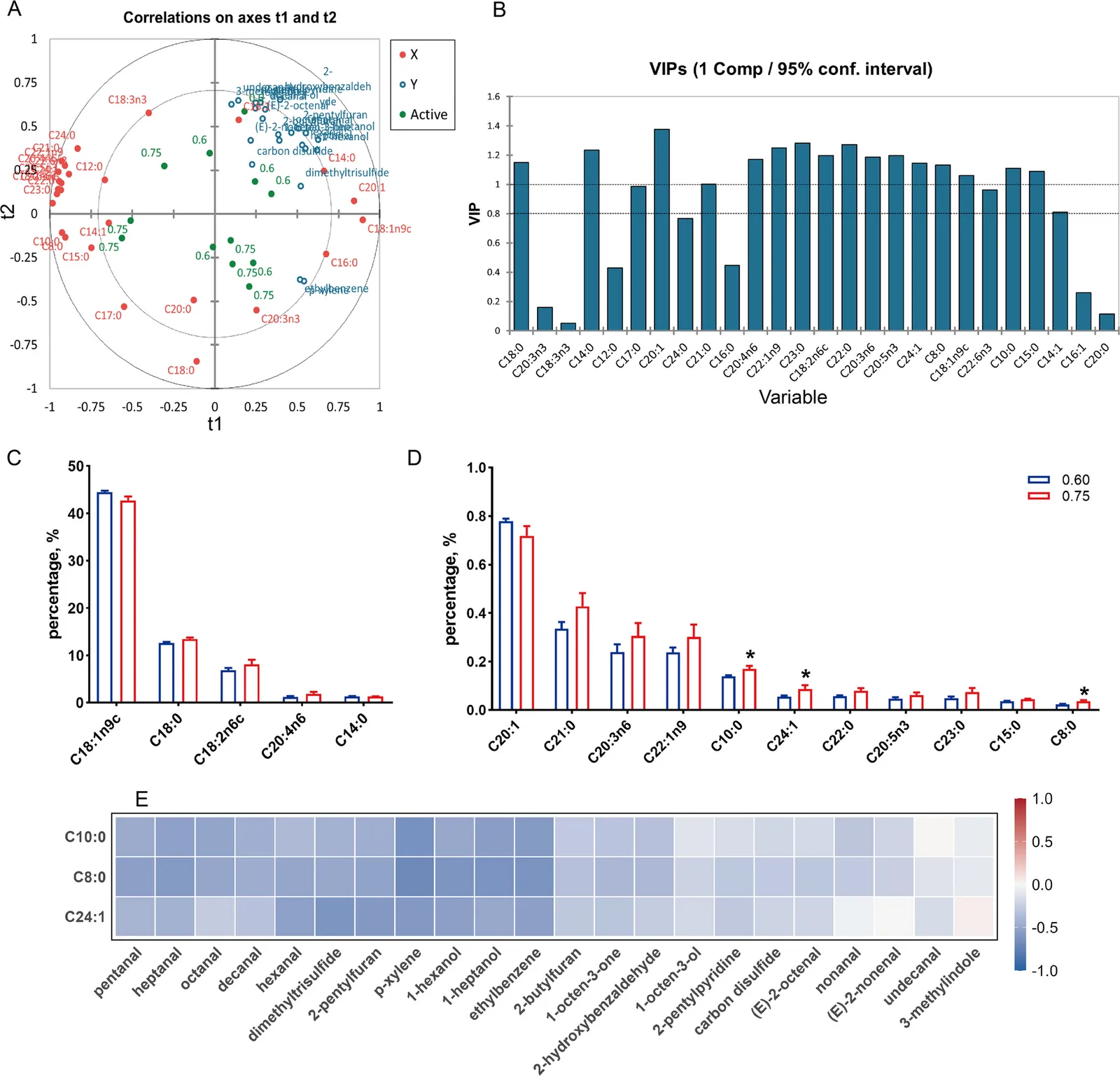
Correlation between volatile organic compounds and fatty acids in castrated fini shing barrows. **A** Loadings plot of the partial least squares regression (PLSR) analysis between volatile organic compounds (*Y* variables) and fatty acids (*X* variables). **B** Potentially important fatty acids with variable importance in projection (VIP) > 1 based on the PLSR model. **C** and **D** Comparison of fatty acids with VIP > 1 between 0.60 and 0.75 groups. **E** Pearson correlation analysis between differential fatty acids and volatile compounds. 0.60, pigs fed diet with P/L ratio = 0.60; 0.75, pigs fed diet with P/L ratio = 0.75. ^*^P < 0.05

Since these 22 VOCs significantly contributed to pork flavor, PLSR was employed to assess the correlations between fatty acid composition and the concentrations of these 22 VOCs in pigs fed diets with different P/L ratios. As shown in Fig. 4A, samples from the 0.60 and 0.75 P/L groups were clearly separated from each other. A total of 16 kinds of fatty acids had VIP scores exceeding 1 (Fig. 4B). The concentrations of C8:0, C10:0, and C24:1 in pork from these two groups showed significant differences (Fig. 4C and D). Furthermore, the concentrations of these fatty acids were negatively correlated with the concentrations of the key pork VOCs (Fig. 4A and E).

However, no significant correlation was observed between differential gut microbiota and the aroma profile in the Mantel test of pigs fed the diets with P/L ratios of 0.60 and 0.75 (Fig. S2B).

## Discussion

As low-protein diets are increasingly adopted in pig farming, and crystalline amino acids such as Lys, Met, Thr, Trp and Ile are commonly supplemented in diets to formulate balanced diets, Phe has emerged as an amino acid requiring closer evaluation to ensure its levels meet the nutritional requirement of pigs. In particular, the impact of dietary Phe levels on carcass traits and meat quality remains unknown.

The pH of pork is a critical indicator, as it is strongly correlated with meat quality [5]. In the present study, we found that the muscle pH_45min_ postmortem increased quadratically as the dietary P/L ratio increased. An increase in pH_45min_ typically indicates slower postmortem glycolysis and less lactate accumulation, which is beneficial for water-holding capacity [31]. Although the 0.90 P/L group showed a numerically lower drip loss than the other groups, the difference was not statistically significant. Thus, under the present conditions, the dietary P/L ratio had a limited effect on water-holding capacity, although this numerical trend may warrant verification in future large-scale studies.

Pork color is the primary factor influencing consumer purchasing decisions. It has been reported that the tolerance limit for color variation in pork at the point of sale is 2.00 [32], while consumers can perceive a total color difference (ΔE) of approximately 1.0 [33]. Based on these findings, we speculate that the increase in a^*^_45min_ (from 15.32 to 16.24) induced by raising the dietary P/L ratio to 0.75 may affect consumer visual perception. Furthermore, in the sensory color evaluation, although the difference between the 0.75 and 0.60 groups was not statistically significant, the 0.75 group still had the highest color score among the four treatment groups. In this study, we evaluated the effect of dietary P/L ratios on meat quality only in castrated males. Female pigs were not included and sex effects need to be investigated in future studies.

Gut microbiota is a key factor influencing gut health and animal production performance. Although phenylalanine is known to be essential for rumen microbial growth in ruminants [34, 35], its influence on swine gut microbiota remains unclear. In this study, dietary phenylalanine supplementation significantly altered the gut microbial composition, with the most pronounced changes observed in pigs fed a diet with a P/L ratio of 0.75. Among these changes, the abundance of the acetate-producing bacterium *NK4A214_group* significantly increased. Notably, acetate has been shown to be crucial for maintaining intestinal immune homeostasis and defending against harmful bacteria [36]. However, in the present study, we did not directly evaluate gut barrier function in pigs fed the diet supplemented with phenylalanine, which warrants further verification.

Substantial evidence confirms that the gut microbiota plays a pivotal role in shaping muscle fatty acid profiles [18, 37, 38]. In the present study, as P/L ratio increased to 0.75, PICRUSt2-predicted functional metabolic pathways of the gut microbiota shifted toward Fatty acid metabolism and Propanoate metabolism. By contrast, at P/L ratio of 0.60, Fructose and mannose metabolism, as well as Amino sugar and nucleotide sugar metabolism, were dominant. These findings suggest that the dietary P/L ratio induced alterations in pork fatty acid profile may be associated with changes in the gut microbiota. Furthermore, the Mantel analysis specifically identified significant correlations between the abundances of *Coprococcus*, *Lachnospiraceae_NK4B4_group*, and *Dialister* and the muscle fatty acid profile. Notably, we observed that the levels of C8:0, C10:0, C22:6n3 and C24:1 were increased in the muscle of pigs fed a diet with a P/L ratio of 0.75. Therefore, we propose that the changes in pork fatty acids at a dietary P/L ratio of 0.75 relative to 0.60 are associated with the concurrent reshaping of the gut microbiota. However, it should be noted that fecal microbiota provides an indirect representation of gut microbial ecology and does not directly measure intestinal metabolic activity. Thus, further validation is required to confirm this associative link.

The inherent flavor precursors of pork, such as fatty acids, amino acids, and nucleotides, constitute fundamental substrates for the formation of flavor compounds during meat cooking [39]. During meat cooking, these precursors engage in the Maillard reaction, thiamine degradation, Strecker degradation, and lipid oxidation, collectively generating a diverse array of VOCs that impart the characteristic aroma to meat [40]. To date, although over 600 VOCs, including aldehydes, ketones, alcohols, phenols, acids, esters, furans, pyrazines, hydrocarbons, and nitrogen/sulfur-containing compounds, have been identified in meat and meat products, only a few with concentrations exceeding their odor thresholds (OAV > 1) can be sensorially perceived and contribute to characteristic flavors [41]. In the present study, we identified 22 compounds with OAV > 1, which are considered to constitute the overall flavor profile of pork. Among these compounds, 1-octen-3-ol (mushroom-like), (E)-2-nonenal (fatty; oily), and hexanal (grassy) are widely recognized as key characteristic aroma compounds in pork [42]. Furthermore, compared with the 0.60 P/L ratio, 12 VOCs were altered when the ratio was increased to 0.75, among which only 2-pentylfuran and decanal had OAVs exceeding 1. It has been reported that 2-pentylfuran and decanal are associated with fatty and buttery notes, respectively [28]. Thus, the reduction in these two compounds may affect these aroma notes. However, given that VOC analysis was limited to the 0.60 and 0.75 groups, further investigation is required to determine the impact of dietary P/L ratio on the sensory flavor perception of pork and its potential dose-dependent effects.

Fatty acids, as crucial flavor precursors, have a decisive influence on the formation of the pork VOC profile [43]. In the present study, we identified 16 fatty acids influencing pork volatile compounds, which are the main precursors for the formation of volatile compounds. Among them, C8:0, C10:0 and C24:1 showed a significant increase in pork from pigs fed a diet with a P/L ratio of 0.75 compared to the ratio of 0.60, and demonstrated a negative correlation with 22 key VOCs. Previous studies have shown a significant correlation between specific fatty acids and volatile compounds [42]. Therefore, we suggest that variations in C8:0, C10:0, and C24:1 were associated with changes in VOC profiles and may be potential contributors, although direct precursor-product relationships require further validation.

Recent studies have indicated a significant correlation between gut microbiota and pork VOCs [44]. However, our finding did not reveal a direct relationship between the two. Emerging evidence indicates that the gut microbiota plays a pivotal role in modulating host fatty acid metabolism and composition [45, 46]. Here, we observed that the gut microbiota was significantly correlated with muscle fatty acid profiles, and these fatty acids were significantly correlated with multiple VOCs.

Taste perception is elicited through the stimulation of gustatory nerve endings by meat-derived flavor compounds, such as inorganic salts, free amino acids, small peptides, and nucleic acid metabolites [47]. Free amino acids are critical flavor compounds that determine the taste profile of pork [9]. In this study, the levels of most free amino acids were significantly increased at the higher P/L ratio of 1.05. This suggested that an excessive P/L ratio disrupts amino acid metabolic homeostasis, leading to the increase in the free amino acid pool [48]. However, the comprehensive effect of simultaneous alterations in multiple amino acid levels on the overall taste profile of pork remains to be further clarified. Furthermore, further studies should incorporate sensory evaluation and include sex as a variable on a larger scale to enhance the robustness of the findings.

## Conclusions

In summary, dietary P/L ratios were associated with alterations in meat quality, free amino acid profile, and fatty acid composition of pork in castrated barrows. Compared with the 0.60, the 0.75 P/L ratio significantly enhanced gut microbial α-diversity (as indicated by the Ace and Chao indices), increased pork contents of C8:0, C10:0, and C24:1, and modulated the composition of VOCs. However, further sensory evaluation is warranted to assess the influence of dietary phenylalanine on meat flavor.

## Supplementary Information

Additional file 1: Fig. S1. Effects of dietary P/L ratio on beta diversity and community composition of fecal microbiota in finishing pigs. Fig. S2. Permutation test for OPLS-DA of VOCs and Mantel’s test between fecal microbiota and VOCs. Table S1. Effects of dietary P/L ratio on the Alpha diversity of fecal microbiota in finishing pigs.

## Data Availability

The datasets used or analysed during the current study are available from the corresponding author on reasonable request.

## Abbreviations

*a*^***^: Redness
*b*^***^: Yellowness
IMF: Intramuscular fat
*L*^***^: Lightness
LT: *Longissimus thoracis*
OAV: Odor activity value
OPLS-DA: Orthogonal Partial Least Squares Discriminant Analysis
PCA: Principal Component Analysis
P/L: Phenylalanine/lysine
PLSR: Partial Least Squares Regression
PUFA: Polyunsaturated fatty acids
SID: Standardized ileal digestible
VIP: Variable importance in projection
VOCs: Volatile organic compounds
