# Supplementary material for: Effects of phenylalanine supplementation on meat fatty acid composition, gut microbiota, and pork volatile organic compound profile in finishing pigs
Source: J Anim Sci Biotechnol. 2026 Sep 12;17:181. doi: 10.1186/s40104-026-01496-7 (PMC13570519; doi:10.1186/s40104-026-01496-7)
Supplement: Supplementary file 1 [file 40104_2026_1496_MOESM1_ESM.docx]

Fig. S1. Effects of dietary P/L ratio on beta diversity and community composition of fecal microbiota in finishing barrows

Beta diversity analysis of fecal microbiota (A), Venn diagram analysis of species (B), and microbial community composition at the phylum (C) and genus (D) levels.

Fig. S2. Permutation test for OPLS-DA of VOCs and Mantel’s test between fecal microbiota and VOCs.

(A) Permutation test for OPLS-DA of VOCs. (B) Mantel’s test between fecal microbiota and VOCs.

**Table S1 Effects of dietary P/L ratio on the Alpha diversity of fecal microbiota in finishing pigs (*n* = 6)**

| Items | Dietary P/L ratio | | | | SEM | *P* value | | |
| --- | --- | --- | --- | --- | --- | --- | --- | --- |
|  | 0.60 | 0.75 | 0.90 | 1.05 |  | Anova | Linear | Quadratic |
| Ace index | 1068.16^b^ | 1291.30^a^ | 1206.62^ab^ | 1195.49^ab^ | 44.47 | 0.02 | 0.15 | 0.02 |
| Chao index | 1038.15^b^ | 1245.32^a^ | 1175.71^ab^ | 1147.23^ab^ | 39.90 | 0.01 | 0.16 | 0.01 |
| Shannon index | 3.48 | 3.95 | 3.91 | 3.86 | 0.18 | 0.24 | 0.17 | 0.16 |
| Simpson index | 0.12 | 0.08 | 0.07 | 0.10 | 0.02 | 0.15 | 0.32 | 0.04 |

^a, b^ Within a row, means without a common superscript differ at *P* < 0.05.
